# Diagnostic accuracy and safety of Diaskintest^®^ compared with the tuberculin skin test for detecting tuberculosis infection in BCG-vaccinated Brazilian adults

**DOI:** 10.3389/fmed.2026.1736211

**Published:** 2026-01-27

**Authors:** Afrânio Kritski, Carla Conceição, Martha Oliveira, Ana Paula Razal Dalvi, Marcelo Cordeiro-Santos, Adriana Rezende Moreira, Aline Lopes, Rogerio Ruffino, Luciana Rodrigues, Eliene Mesquita, Sergian Vianna Cardozo, Maria Jose Fernandes Pereira, Sumire Sakabe, Anete Trajman, Marcela Bhering

**Affiliations:** 1 Hospital Universitário Clementino Fraga Filho/Instituto de Doenças do Tórax, Escola de Medicina, Universidade Federal do Rio de Janeiro, Rio de Janeiro, Brazil; 2 Laboratório de Micobacteriologia Molecular, Universidade Federal do Rio de Janeiro, Rio de Janeiro, Brazil; 3 Fundação de Medicina Tropical Dr. Heitor Vieira Dourado, Manaus, Brazil; 4 School of Medicine, Universidade do Estado do Amazonas, Manaus, Brazil; 5 School of Medicine, Universidade Nilton Lins, Manaus, Brazil; 6 Centro Municipal de Saúde de Duque de Caxias, Municipal Health Secretariat, Duque de Caxias, Brazil; 7 Hospital Universitário Pedro Ernesto, Universidade do Estado do Rio de Janeiro, Rio de Janeiro, Brazil; 8 Instituto Ary Parreiras State, State Health Secretariat of Rio de Janeiro, Niterói, Brazil; 9 School of Medicine, Universidade do Grande Rio Professor José de Souza Herdy, Duque de Caxias, Brazil; 10 Serviço de Atenção Especializada em Tuberculose, Municipal Health Secretariat of Itaboraí, Itaboraí, Brazil; 11 Centro de Referência e Treinamento DST-AIDS-SP, São Paulo, Brazil; 12 Centro de Referência Professor Hélio Fraga, Escola Nacional de Saúde Pública, Fundação Oswaldo Cruz, Rio de Janeiro, Brazil

**Keywords:** diagnosis, latent tuberculosis infection, recombinant protein, tuberculin, tuberculosis, tuberculosis-specific skin tests

## Abstract

**Background:**

Evidence on the accuracy and safety of the ESAT-6/CFP-10–based Diaskintest^®^ in BCG-vaccinated populations outside Eastern Europe remains limited. In Brazil, recurrent shortages of purified protein derivative (PPD) have challenged the implementation of tuberculin skin testing, underscoring the need to evaluate alternative tools for tuberculosis infection (TBI) screening. This trial compared the diagnostic performance and safety of Diaskintest^®^ with the tuberculin skin test (TST) using PPD Rt-23 in Brazilian adults, a predominantly BCG-vaccinated population.

**Method:**

A double-blind randomized clinical trial was conducted at eight centers in Brazil between July 2023 and September 2024. Participants were allocated to the TB group (microbiologically confirmed pulmonary TB), in whom sensitivity was estimated, and the control group (healthy, unexposed adults), in whom specificity was estimated using the QuantiFERON-TB Plus^®^ (QFT-Plus) as the reference standard. All participants first underwent QFT-Plus testing, followed by intradermal application of Diaskintest^®^ and TST in opposite arms, with randomized right–left allocation. Induration was measured at 48–96 h using a prespecified 5 mm cutoff. Secondary outcomes included safety, assessed through active monitoring of adverse events (AEs), included injection site reactions.

**Results:**

A total of 337 controls and 136 TB participants were enrolled. TST showed higher sensitivity than both Diaskintest and QFT-plus (0.84 [95% CI 0.76–0.90] vs. 0.68 [95% CI 0.59–0.76], and 0.63 [95% CI 0.54–0.72], respectively). Diaskintest^®^ demonstrated higher specificity than TST (0.93 [95% CI 0.90–0.96] vs. 0.75 [95% CI 0.70–0.80]). Injection-site reactions occurred less frequently with Diaskintest^®^ than with TST (1.7% vs. 4.9%. RR = 0.35 [0.15–0.79]). The most common reactions were phlyctenular reactions and itching. No serious AEs were observed.

**Conclusion:**

TST had greater sensitivity than Diaskintest^®^, whereas Diaskintest^®^ demonstrated higher specificity and fewer local adverse reactions. In this study, specificity was estimated using QFT-Plus as a surrogate reference standard, acknowledging the absence of a true gold standard for tuberculosis infection. These complementary performance profiles highlight a trade-off between false-positive reduction and case detection, suggesting that the choice of test should consider programmatic priorities and local epidemiological context.

**Clinical trial registration:**

https://ensaiosclinicos.gov.br/, identifier RBR-7tn2ysw.

## 1 Introduction

Tuberculosis (TB), caused by *Mycobacterium tuberculosis* (MTB), remains a major global public health challenge. Approximately one quarter of the world’s population is infected with MTB, and the lifetime risk of progression to active TB varies widely depending on host factors, including HIV status and immunosuppression, but is estimated at around 5–10% in the general population (1). This large reservoir of infection can sustain transmission for decades if not identified and treated. Thus, TB preventive treatment (TPT) is a cornerstone strategy for disease control and eventual elimination (2).

For several decades, tuberculosis infection (TBI) has been diagnosed using the tuberculin skin tests (TST), performed with purified protein derivatives (PPDs) (3). However, the TST has important conceptual limitations. Cross-reactivity with Bacillus Calmette-Guérin (BCG) vaccination and non-tuberculous mycobacteria (NTM) may lead to false-positives results, while false-negative reactions can occur in individuals with advanced HIV disease, malnutrition, severe TB, or impaired cellular immunity (4, 5). In parallel, operational challenges, particularly recurrent global shortages of PPD Rt-23 (AJ Vaccines, Copenhagen, Denmark) over the past two decades, have hindered TBI screening in high-risk groups such as people living with HIV (PLHIV), household contacts, and high-risk occupational populations. These supply interruptions have affected clinical practice in countries like Brazil, where TBI testing is routinely recommended (6).

In 2011, the World Health Organization (WHO) endorsed interferon-gamma release assays (IGRAs) for TBI diagnosis (3). Because they use MTB*-*specific antigens, IGRAs generally offer higher specificity than the TST (7). However, their implementation requires laboratory infrastructure, stringent processing times, reliable electricity, and skilled personnel. These constraints significantly limit their feasibility in resource-limited settings, including many regions of Brazil, where decentralized services and primary care networks have limited capacity to perform blood-based assays (8).

To overcome these challenges, newer tuberculosis-specific skin tests (TBSTs) were developed using recombinant MTB-specific antigens such as ESAT6 and CFP10 (9). A systematic review and meta-analysis of 37 studies reported that TBST accuracy was comparable to that of IGRAs, with consistent performance across different populations, including children, adults, and PLHIV (10). Across seven studies reporting safety outcomes, profiles were similar to TST, with mostly mild-to-moderate injection site reactions. In 2022, WHO issued a conditional recommendation for the use of three TBSTs (Cy-Tb®, Diaskintest®, and C-TST) based on moderate-certainty evidence and their potential applicability in high TB-burden settings (11).

Despite these developments, most evaluations of Diaskintest® and C-TST have been conducted in China and Eastern European countries, with limited evidence from high-burden countries outside these regions. Moreover, few studies adhered fully to elements of the WHO’s 2020 Framework for new TBI tests, which emphasizes the need for appropriate inclusion of at-risk populations, harmonized reference standards, and rigorous assessment of safety and operational feasibility (12). Importantly, no trial has assessed the accuracy and safety of Diaskintest® in BCG-vaccinated adults in Latin America, a key evidence gap given the near-universal neonatal BCG vaccination policy in countries such as Brazil.

Diaskintest®, which uses ESAT-6 and CFP-10 antigens, is of particular interest in this context because it is expected to maintain high specificity in BCG-vaccinated populations, is administered using the same technique as TST, and may be more operationally feasible than IGRAs in resource-constrained settings. Evaluating its performance in Brazil is therefore timely and highly relevant for informing national diagnostic strategies.

Brazil is one of the 30 TB high-burden countries, with an estimated TB/HIV co-infection rate of 9.3% in 2023 (13, 14). Neonatal BCG vaccination has been recommended nationwide for decades, with coverage historically above 95% (15, 16). These contextual characteristics highlight the need to assess diagnostic tools that preserve high specificity in BCG-vaccinated populations while remaining feasible for programmatic implementation.

We therefore conducted a clinical trial to evaluate the accuracy and safety of Diaskintest® for the diagnosis of tuberculosis infection, compared with the tuberculin skin test (TST) and QFT-Plus (Qiagen, Hilden, Germany), across eight health units in different regions of Brazil.

## 2 Materials and methods

### 2.1 Study design

This is a randomized, double-blinded clinical trial comparing the diagnostic accuracy and safety of Diaskintest® with TST (performed with PPD Rt-23) and QuantiFERON-TB Plus® (QFT-Plus). Sensitivity was assessed among adults with microbiologically confirmed tuberculosis, whereas specificity was assessed among healthy, unexposed adults.

### 2.2 Study setting

The study was conducted between July 2023 and September 2024 across eight health units located in the states of Rio de Janeiro, São Paulo, and Amazonas. The centers were selected to represent the primary care and referral facilities in which TBI testing is routinely performed in Brazil, including both specialized TB services and high-volume outpatient units. This selection ensured representation of diverse epidemiological contexts and operational conditions relevant to national TBI screening strategies.

### 2.3 Study population

Participants aged 18 to 59 years were recruited into two groups: TB Group: adults with active pulmonary TB confirmed by Xpert MTB/RIF ULTRA® and/or positive MTB culture, at any stage of anti-TB treatment; Control Group: asymptomatic adults with no known exposure to TB and no history of active TB or prior positive TST.

Participants were excluded if they had: (i) conditions known to impair the interpretation of delayed-type hypersensitivity responses (e.g., severe malnutrition, current use of immunosuppressive therapy); (ii) known or suspected infection with NTM, due to potential cross-reactive immune responses; (iii) a documented or self-reported history of exaggerated reaction to previous TST (e.g., phlyctenular reaction); (iv) pregnancy or breastfeeding, based on ethical considerations related to investigational diagnostic procedures.

For the control group specifically, individuals with prior TB, prior positive TST, or any suggestion of TB exposure were excluded. All participants provided written informed consent.

Losses were defined as individuals who consented but were subsequently excluded due to eligibility issues, withdrawal, or failure to complete required assessments. Participants in the TB Group who did not return for the skin-test reading but had valid QFT-Plus results were included in the sensitivity analysis for QFT-Plus only.

### 2.4 Outcome measures

The primary outcomes were the sensitivity and specificity of Diaskintest® compared with TST using a 5 mm positivity cutoff. This threshold follows Brazilian national recommendations for adults at increased risk of progression, including people living with HIV, who represented an important share of our cohort, and has also been used in previous clinical evaluations of Diaskintest®. Higher cutoffs (10 mm and 15 mm) were explored in secondary analyses (17–19).

Secondary outcomes included diagnostic accuracy stratified by age, HIV status, and BCG vaccination, as well as the frequency and severity of adverse events.

### 2.5 Sample size

Primary outcomes were sensitivity and specificity of the Diaskintest® compared with TST using a 5 mm cutoff. Based on a systematic review, we assumed a TST specificity of 59% in BCG-vaccinated populations and QFT-Plus specificity of 95% (20). To detect a minimum 5-percentage-point improvement in specificity with 80% power and a 0.05% significance level, 316 participants were required in the control group.

For sensitivity analyses, assuming 80% for TST and a 10% non-inferiority margin, 137 participants with active TB were needed to achieve 80% power at a 0.05 significance level.

Secondary outcomes included safety events and diagnostic accuracy stratified by age, HIV status, and BCG vaccination.

### 2.6 Intervention

After written informed consent, sociodemographic and clinical information was collected. BCG vaccination was assessed by documentation and/or presence of a BCG scar. HIV testing was offered but not mandatory. Women underwent a urine pregnancy test.

All participants underwent phlebotomy for QFT-Plus prior to skin-test administration.

Diaskintest and TST were administered intradermally following the Mantoux technique (21). The tests were applied in opposite arms, with randomization determining right–left allocation (1:1). This design minimized the possibility of local immunological interaction or order effects, as delayed-type hypersensitivity responses do not cross between limbs.

Induration (recorded in millimeters) was measured by trained, blinded staff between 48–96 h, consistent with operational standards in outpatient practice (Supplementary Figure S1). Although 72 h is the most common reference point, readings up to 96 h are acceptable and were used to maximize participant follow-up (21).

The blinding procedure consisted of one team member preparing the syringes labeled only as right or left, while another, unaware of the allocation or QFT-Plus results, performed the application and reading.

QFT-Plus samples were processed according to manufacturer instructions. Indeterminate results were retested once following repeat venous blood collection.

### 2.7 Adverse event evaluation

Adverse events (AEs) were categorized as injection site reactions (ISRs), including pain, itching, phlyctenule or hyperpigmentation (19). These were graded by intensity (mild/moderate/severe) and seriousness according to Brazilian regulatory criteria (22). A serious AE was defined as any event resulting in significant clinical consequences, irrespective of causality.

AEs were assessed immediately after test administration, at the reading visit, and through participant-initiated reporting for up to 1 month. Follow-up included telemonitoring (video or photographic documentation) or in-person evaluation when necessary.

### 2.8 Analyses

Descriptive statistics summarized baseline characteristics of the participants by group (TB vs. control), including sex, age, HIV status, CD4 count, and BCG vaccination. QFT-Plus results were classified as positive, negative, or indeterminate. CD4 counts were not collected as part of the study protocol; when available, they were retrieved from routine clinical records within the Brazilian Unified Health System (SUS), and were therefore available only for a subset of participants living with HIV.

For both Diaskintest® and TST, induration was categorized as negative (0–4 mm) or positive (≥5 mm) in the primary analyses. Sensitivity was calculated as the proportion of positive results among participants with microbiologically confirmed TB; and specificity as the proportion of negative results among controls.

Sensitivity of TST, Diaskintest®, and QFT-Plus was estimated among participants with confirmed TB. Specificity for both skin tests was estimated among controls using QFT-Plus as the reference standard. Secondary analyses evaluated accuracy at 10 mm, and 15 mm cutoffs and stratified results by age, HIV status, and BCG vaccination.

Adverse events were analyzed by test and group. Risk ratios (RR) with 95% confidence intervals (CI) compared injection-site reaction frequency between Diaskintest® and TST. Paired proportions were compared using McNemar’s test.

## 3 Results

### 3.1 Sample characteristics and overall screening results

**Figure 1:**
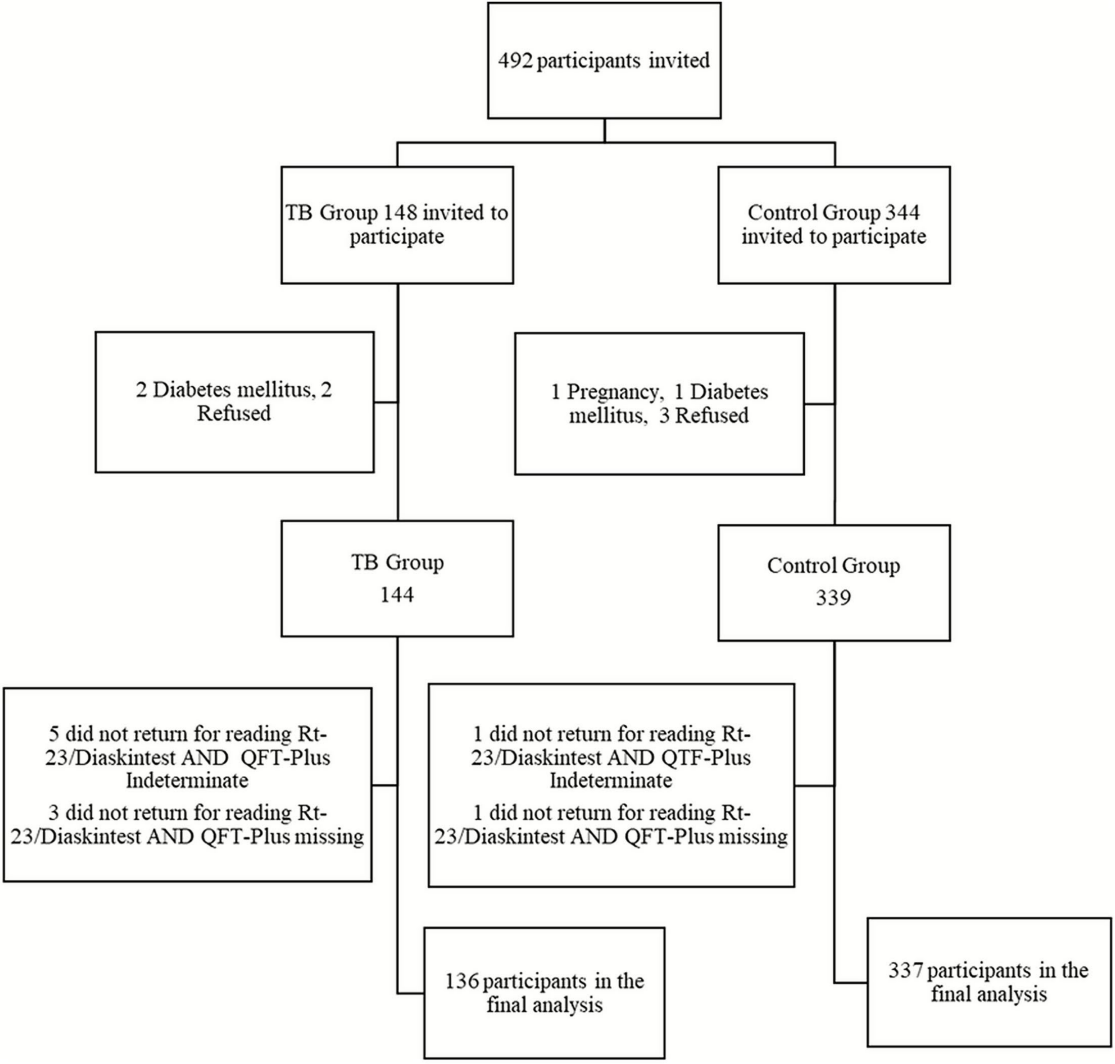
Flowchart of participants through the study.

Between July 2023 and September 2024, 492 individuals were assessed for eligibility. After exclusions and losses (Figure 1), 473 participants were enrolled in the final analysis: 136 in the TB group and 337 in the control group.

Table 1 summarizes baseline characteristics. In the TB group, 30/136 (22.1%) were female and 41/129 (31.8%) were PLHIV. CD4 counts were available from routine SUS clinical records for 28/41 PLHIV in the TB group and 13/34 PLHIV in the control group; among PLHIV with available data, the median CD4 count in the TB group was 151 cells/μL (IQR 94.7–380.2). BCG vaccination was documented or evidenced by scar in 128/136 (94.1%). Participants in the control group were predominantly female (218/337; 64.6%) and younger, with 159/337 (47.1%) aged 18–24 years. Among those tested for HIV, 34/253 (13.4%) were PLHIV. Most controls were BCG-vaccinated (330/337; 97.9%).

**Table 1:** Sociodemographic and clinical characteristics of participants.

Characteristics	TB group *N* = 136	Control group *N* = 337
Sex		
Female	30 (22.1%)	218 (64.7%)
Male	106 (77.9%)	119 (35.3%)
Age group		
18–24 years	21 (15.5%)	159 (47.2%)
25–39 years	63 (46.3%)	128 (38.0%)
40 years or more	52 (38.2%)	50 (14.8%)
Body Mass Index, *N* = 136/333		
Less than 18.5	55 (40.4%)	13 (3.9%)
18.5–24.9	72 (53.0%)	168 (50.5%)
25–29.9	6 (4.4%)	91 (27.3%)
≥30	3 (2.2%)	61 (18.3%)
HIV status, *N* = 129/253		
Negative	88 (64.8%)	219 (65.0%)
Positive	41 (30.1%)	34 (10.1%)
CD4 count (cells/μL), *N* = 28/13		
Median (IQR)	151 (94.7–380.2)	691 (429–849)
BCG scar		
No	8 (5.9%)	7 (2.1%)
Yes	128 (94.1%)	330 (97.9%)

TB, tuberculosis; HIV, human immunodeficiency virus; BCG, Bacillus Calmette-Guérin; IQR, Interquartile Range; CD4, Cluster of Differentiation 4.

Valid Diaskintest® and TST results were available for 429/473 (90.7%) participants, while QFT-Plus results were available for 468/473 (98.9%). QFT-Plus initially yielded 26 indeterminate results, which were retested using a newly collected blood sample; after repeat testing, 26/468 (5.6%) results remained indeterminate, and 15/473 (3.1%) were missing due to laboratory processing issues (Table 2).

**Table 2:** Results of PPD RT23, Diaskintest, and QFT-Plus by group.

Results	QFT-plus result							
	Positive		Negative		Indeterminate		Missing	
	TB group *N* = 71	Control group *N* = 35	TB group *N* = 41	Control group *N* = 295	TB group *N* = 18	Control group *N* = 8	TB group *N* = 14	Control group, *N* = 1
PPD RT23								
Negative	12 (20.3%)	13 (39.4%)	5 (15.2%)	226 (82.8%)	1 (7.7%)	5 (71.4%)	1 (9.1%)	0
Positive	47 (79.7%)	20 (60.6%)	28 (84.8%)	47 (17.2%)	12 (92.3%)	2 (28.6%)	10 (90.9%)	0
Missing	12 (16.9%)	2 (5.7%)	8 (19.5%)	22 (7.5%)	5 (27.8%)	1 (12.5%)	3 (21.4)	1 (100%)
Diaskin								
Negative	14 (23.7%)	18 (54.6%)	15 (45.4%)	270 (98.9%)	5 (38.5%)	6 (85.7%)	3 (27.3%)	0
Positive	45 (76.3%)	15 (45.4%)	18 (54.6%)	3 (1.1%)	8 (61.5%)	1 (14.3%)	8 (72.7%)	0
Missing	12 (16.9%)	2 (5.7%)	8 (19.5%)	22 (7.5%)	5 (27.8%)	1 (12.5%)	3 (21.4%)	1 (100%)

### 3.2 Primary outcome

At the 5 mm cutoff, among participants with microbiologically confirmed TB, TST showed higher sensitivity (0.84; 95% CI 0.76–0.90) than Diaskintest® (0.68; 95% CI 0.59–0.76) and QFT-Plus (0.63; 95% CI 0.54–0.72) (Table 3). The difference between TST and Diaskintest® was statistically significant (*p* < 0.01). Sensitivity decreased for both skin tests at 10 mm and 15 mm cutoffs; the superiority of TST remained significant at 10 mm but not at 15 mm.

**Table 3:** Sensitivity (TB group) and specificity (controls; QFT-Plus reference) of Diaskintest and TST at different cutoff points.

Cutoff points (millimetres)	Sensitivity Diaskintest® (95% CI)	Sensitivity TST (95% CI)	Sensitivity QFT-Plus* (95% CI)	Specificity Diaskintest® (95% CI)	Specificity TST (95% CI)
5	0.68 (0.59–0.76)	0.84 (0.76–0.90)	0.63 (0.54–0.72)	0.93 (0.90–0.96)	0.75 (0.70–0.80)
10	0.59 (0.49–0.68)	0.72 (0.62–0.80)	_	0.95 (0.92–0.97)	0.85 (0.80–0.88)
15	0.43 (0.34–0.53)	0.50 (0.41–0.59)	_	0.96 (0.94–0.98)	0.93 (0.89–0.95)

*QFT-Plus values refer only to sensitivity analyses (tested among participants with microbiologically confirmed TB).

Specificity in the control group showed the opposite pattern (Table 3). At the 5 mm cutoff, Diaskintest® demonstrated markedly higher specificity (0.93; 95% CI 0.90–0.96) than TST (0.75; 95% CI 0.70–0.80). This difference remained statistically significant at the 10 mm but not at 15 mm, when both tests reached similarly high specificity values.

### 3.3 Secondary outcomes

#### 3.3.1 Subgroup analyses

Across all age groups, the performance patterns observed in the primary analysis were preserved. Among participants aged 18–24 years (Supplementary Figure S2), TST showed higher sensitivity than Diaskintest® at the 5-mm cutoff (0.89 vs. 0.67), whereas Diaskintest® demonstrated higher specificity (0.96 vs. 0.86). QFT-Plus sensitivity in this age group was 0.68 overall, 0.75 among PLHIV, and 0.68 among BCG-vaccinated participants (Supplementary Figure S3). These patterns were consistent at higher cutoffs (10 mm and 15 mm), with Diaskintest® maintaining specificity above 95% across thresholds.

Among participants aged 25–39 years (Supplementary Figure S4), sensitivity at the 5 mm cutoff was higher for TST (0.84) than for Diaskintest® (0.72), while specificity remained higher for Diaskintest® (0.92 vs. 0.66). QFT-Plus sensitivity in this age group was 0.68 overall, increasing to 0.82 among PLHIV and 0.69 among BCG-vaccinated individuals (Supplementary Figure S5). At higher cutoffs, sensitivity declined for both skin tests, whereas Diaskintest® retained specificity above 90%.

In adults aged 40 years or older (Supplementary Figure S6), sensitivity was lower overall. At the 5 mm cutoff, TST sensitivity was 0.80 and Diaskintest® 0.63, while Diaskintest® maintained higher specificity than TST (0.87 vs. 0.67). QFT-Plus sensitivity in this age group was 0.56 overall, 0.67 among PLHIV, and 0.62 among BCG-vaccinated individuals (Supplementary Figure S7). Increasing the cutoff to 10 mm or 15 mm further reduced sensitivity for both skin tests.

Among PLHIV (Supplementary Figure S8), sensitivity at the 5-mm cutoff was reduced for all tests: 0.67 for TST, 0.59 for Diaskintest®, and 0.76 for QFT-Plus. These differences were not statistically significant. Among HIV-negative individuals (Supplementary Figure S9), specificity remained high for both skin tests, with Diaskintest® consistently exceeding 93% across cutoffs. Analyses were based on available HIV data (listwise exclusion for missing values).

BCG vaccination status also influenced diagnostic accuracy. Among BCG-vaccinated participants, who represented nearly the entire study population, TST showed higher sensitivity than Diaskintest® at all cutoffs (5 mm: 0.86 vs. 0.70; 10 mm: 0.74 vs. 0.61; 15 mm: 0.51 vs. 0.45), whereas Diaskintest® consistently demonstrated higher specificity (5 mm: 0.94 vs. 0.76; 10 mm: 0.95 vs. 0.85; 15 mm: 0.97 vs. 0.93). Only 15 participants in the study were not BCG-vaccinated, resulting in imprecise estimates for this subgroup.

#### 3.3.2 Safety

Among the 473 participants, 31 (6.5%) reported injection-site reactions: 8/473 (1.7%) after Diaskintest and 23/473 (4.9%) after TST, corresponding to a risk ratio of 0.35 (95% CI 0.15–0.79) (Table 4). ISRs were more frequent among participants with TB than among controls for both tests. The most common ISR was phlyctenular reaction (41.9%), followed by itching (25.8%). Most reactions were mild (51.6%), and topical treatment was required in 9 participants (1.9%). No serious adverse events or deaths were observed.

**Table 4:** Injection skin events associated with Diaskintest and PPD Rt-23 in TB and control groups.

Injection skin reactions/Groups	Diaskintest		PPD Rt-23		Total	RR* (95% CI)
	TB group *N* (%)	Control group *N* (%)	TB group *N* (%)	Control group *N* (%)		
Type of injection skin reactions						
Phlyctenule	-	1 (3.2)	1 (3.2)	11 (35.5)	13 (41.9)	
Vesicles	1 (3.2)	1 (3.2)	1 (3.2)	1 (3.2)	4 (12.9)	
Itching	2 (6.4)	1 (3.2)	4 (12.9)	1 (3.2)	8 (25.8)	
Erythema	1 (3.2)	-	2 (6.4)	-	3 (9.7)	
Local oedema	1 (3.2)	-	1 (3.2)	-	2 (6.5)	
Pain	-	-	1 (3.2)	-	1 (3.2)	
Total	**5 (16.1)**	**3 (9.7)**	**10 (32.3)**	**13 (41.9)**	**31 (100.0)**	**0.35 (0.15–0.79)**
Intensity						
Mild	1 (3.2)	3 (9.7)	5 (16.1)	7 (22.6)	16 (51.6)	
Moderate	4 (12.9)	-	5 (16.1)	6 (19.3)	15 (48.4)	
Severe	-	-		-	-	
Total	**5 (16.1)**	**3 (9.7)**	**10 (32.3)**	**13 (41.9)**	**31 (100.0)**	

*Relative risk of overall injection skin reactions with Diaskintest compared to PPD Rt-23. Bold values indicate totals.

## 4 Discussion

Most existing evidence on Diaskintest® originates from Russia and neighboring countries (10, 18), where epidemiological and immunological contexts differ substantially from Brazil (13). In those settings, Diaskintest® has consistently shown very high sensitivity (≈90–97%) and specificity exceeding 96% in pediatric and adult populations (18, 19, 23). These differences include historically widespread BCG revaccination policies, the use of distinct BCG strains (24, 25), and different HIV co-infection prevalence (26). By contrast, Brazil applies a single-dose neonatal BCG schedule, uses the BCG Moreau strain, and has a higher burden of HIV co-infection (13, 25). Such factors may influence host immune responses to tuberculin and ESAT-6/CFP-10 antigens and contribute to differences in diagnostic test performance across settings (20).

At the prespecified 5-mm cutoff, aligned with Brazilian recommendations for adults at increased risk, including people living with HIV (17), Diaskintest® showed lower sensitivity than TST and QFT-Plus, while maintaining substantially higher specificity than TST (0.93 vs. 0.75). Increasing the induration cutoff led to the expected reduction in sensitivity for both skin tests, consistent with prior evidence on tuberculin testing (27). However, the relative pattern of higher sensitivity for TST and higher specificity for Diaskintest® was preserved across thresholds. Given the epidemiological profile of our population, including a substantial burden of HIV, the 5-mm threshold remains clinically appropriate for primary analyses. Given the epidemiological profile of our population, including substantial HIV burden, the 5-mm threshold remains clinically appropriate for primary analyses.

Subgroup analyses corroborated the greater dependence of Diaskintest® and QFT-Plus on preserved T-cell-mediated immunity. Among PLHIV, sensitivity declined more markedly for both ESAT-6/CFP-10–based tests than for TST, consistent with their shared antigenic targets and the documented vulnerability of RD1-specific immune responses to immunosuppression (20). TST showed a smaller decline, likely reflecting its broader antigen repertoire, which may elicit detectable delayed-type hypersensitivity responses even with partial immune dysfunction (28). Accuracy patterns across age groups were stable, though sensitivity tended to be lower in older adults, consistent with age-related declines in cell-mediated immunity described in previous literature (29, 30).

Both skin tests demonstrated a favorable safety profile, with significantly fewer injection-site reactions observed for Diaskintest®. Most reactions were mild, and no serious adverse events occurred. These findings are consistent with systematic review evidence indicating that TB antigen–based skin tests have reactogenicity comparable to or lower than that of the TST, with reactions predominantly mild and self-limited (10, 31).

This study has limitations. Specificity was estimated using QFT-Plus as the reference standard. Although endorsed for this purpose, QFT-Plus is an imperfect proxy for true infection status, particularly in immunosuppressed populations or in the presence of indeterminate results and may introduce misclassification. Chest radiography was not performed among asymptomatic QFT-positive controls, so subclinical disease cannot be fully excluded. Subgroup analyses were underpowered, especially among the small number of BCG-unvaccinated participants. In addition, systemic adverse events could not be attributed to a specific test because both skin tests were administered simultaneously.

Despite these limitations, this study provides high-quality evidence from a context characterized by near-universal neonatal BCG vaccination and a high prevalence of HIV, conditions underrepresented in prior Diaskintest® evaluations. Diaskintest® demonstrated higher specificity and fewer local adverse reactions than TST, at the cost of lower sensitivity. These complementary performance characteristics are important for TB programs to consider when the role of ESAT-6/CFP-10-based tests in infection screening strategies.

## 5 Conclusion

Diaskintest® offers higher specificity and a more favorable local safety profile compared with TST using PPD Rt-23, but with consistently lower sensitivity. This trade-off between reducing false positives and preserving case detection is central to TB infection screening policies. Programmatic use of either test should be guided by local epidemiology and screening priorities.

## Data Availability

The raw data supporting the conclusions of this article will be made available by the authors, without undue reservation.

## Supplementary material

SUPPLEMENTARY FIGURE S1 Study protocol.

SUPPLEMENTARY FIGURE S2 Accuracy of Diaskintest and TST among participants aged 18–24 years.

SUPPLEMENTARY FIGURE S3 Accuracy of Diaskintest and TST among participants aged 25–39 years.

SUPPLEMENTARY FIGURE S4 Accuracy of Diaskintest and TST among participants aged 40 years or older.

SUPPLEMENTARY FIGURE S5 Accuracy of QFT-Plus among participants aged 18–24 years.

SUPPLEMENTARY FIGURE S6 Accuracy of QFT-Plus among participants aged 25–39 years.

SUPPLEMENTARY FIGURE S7 Accuracy of QFT-Plus among participants aged 40 years or older.

SUPPLEMENTARY FIGURE S8 Accuracy of Diaskintest and PPD Rt-23 among HIV-positive participants.

SUPPLEMENTARY FIGURE S9 Accuracy of Diaskintest and PPD Rt-23 among HIV-negative participants.
