# Supplementary figures and images for: Diagnostic accuracy and safety of Diaskintest® compared with the tuberculin skin test for detecting tuberculosis infection in BCG-vaccinated Brazilian adults
Source: Front Med (Lausanne). 2026 Jan 27;13:1736211. doi: 10.3389/fmed.2026.1736211 (PMC12886023; doi:10.3389/fmed.2026.1736211)

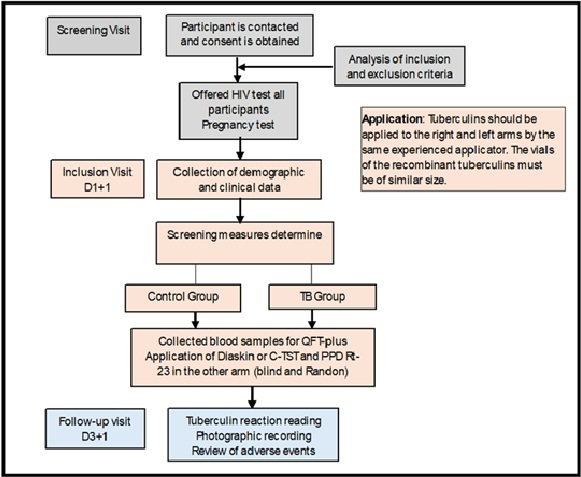

Supplement: SUPPLEMENTARY FIGURE S1 — Study protocol. [file Image_1.TIF]

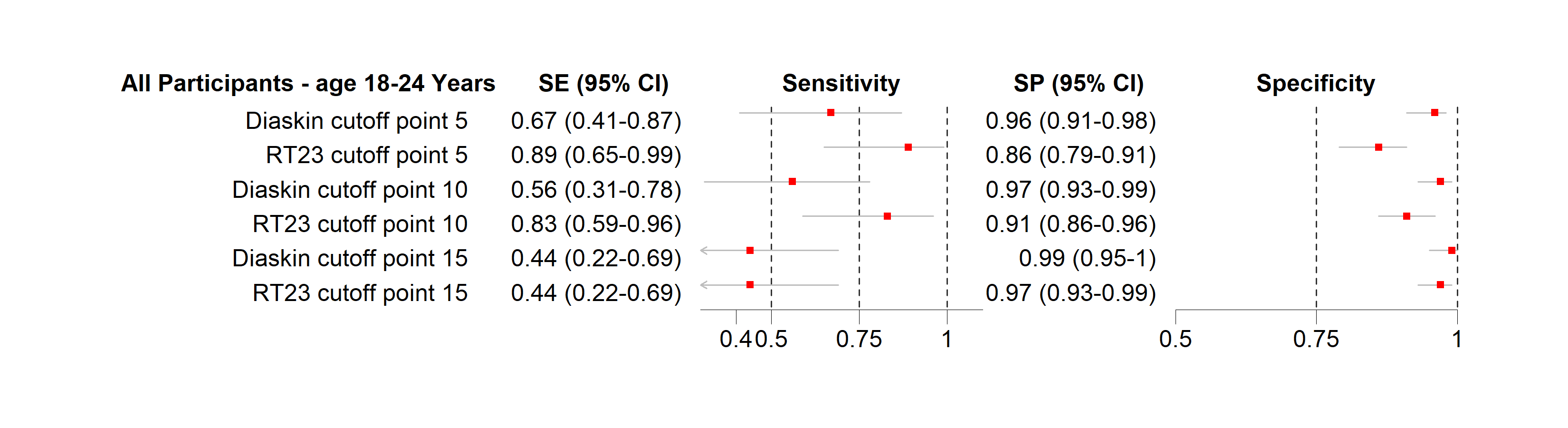

Supplement: SUPPLEMENTARY FIGURE S2 — Accuracy of Diaskintest and TST among participants aged 18–24 years. [file Image_2.TIFF]

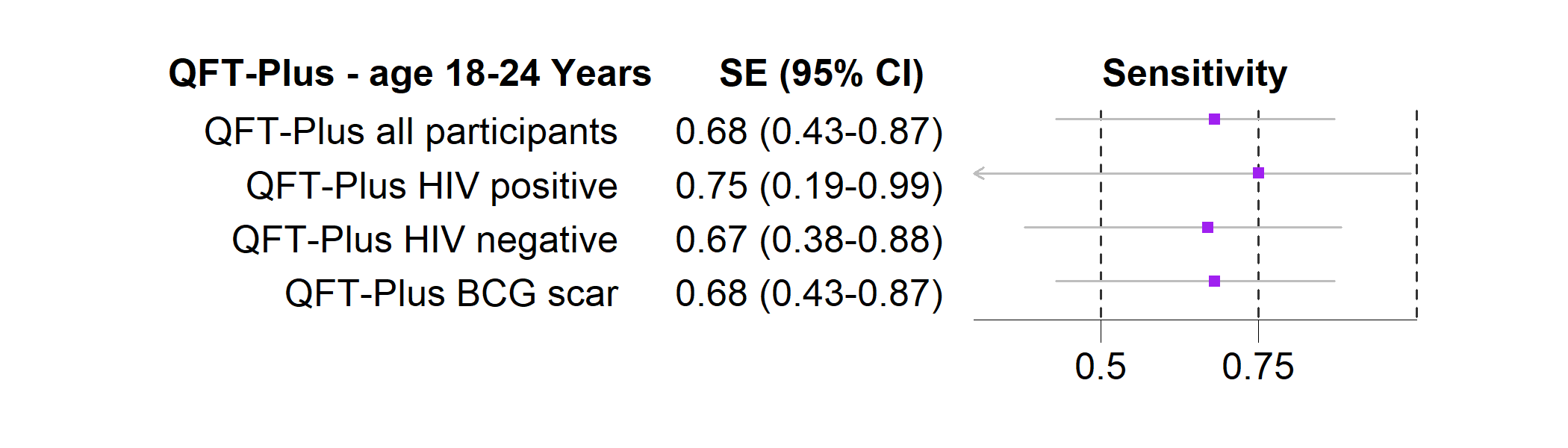

Supplement: SUPPLEMENTARY FIGURE S3 — Accuracy of Diaskintest and TST among participants aged 25–39 years. [file Image_3.TIFF]

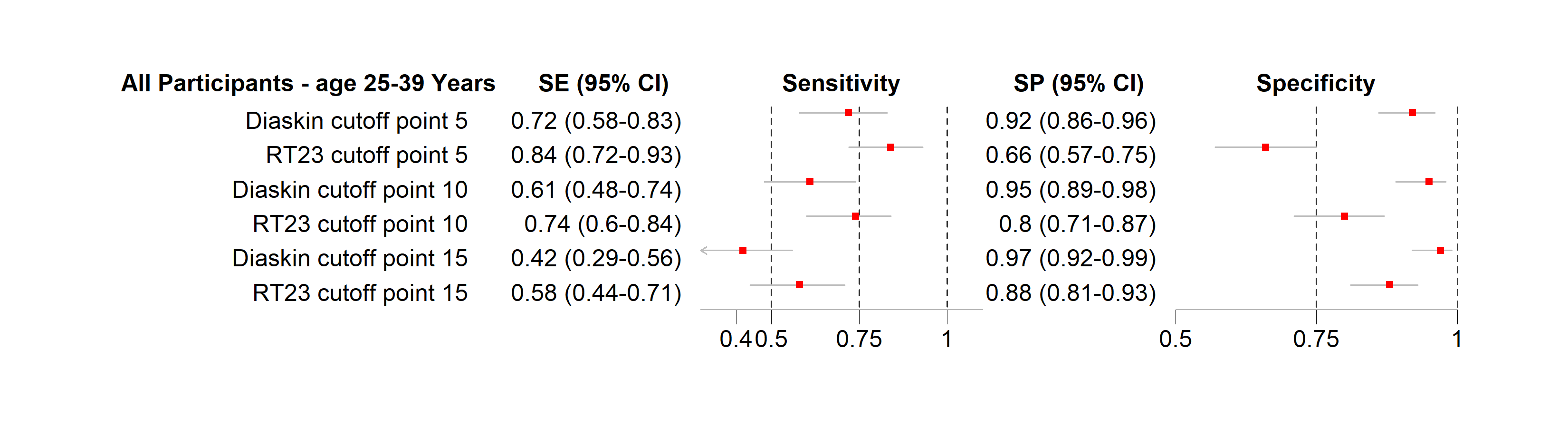

Supplement: SUPPLEMENTARY FIGURE S4 — Accuracy of Diaskintest and TST among participants aged 40 years or older. [file Image_4.TIFF]

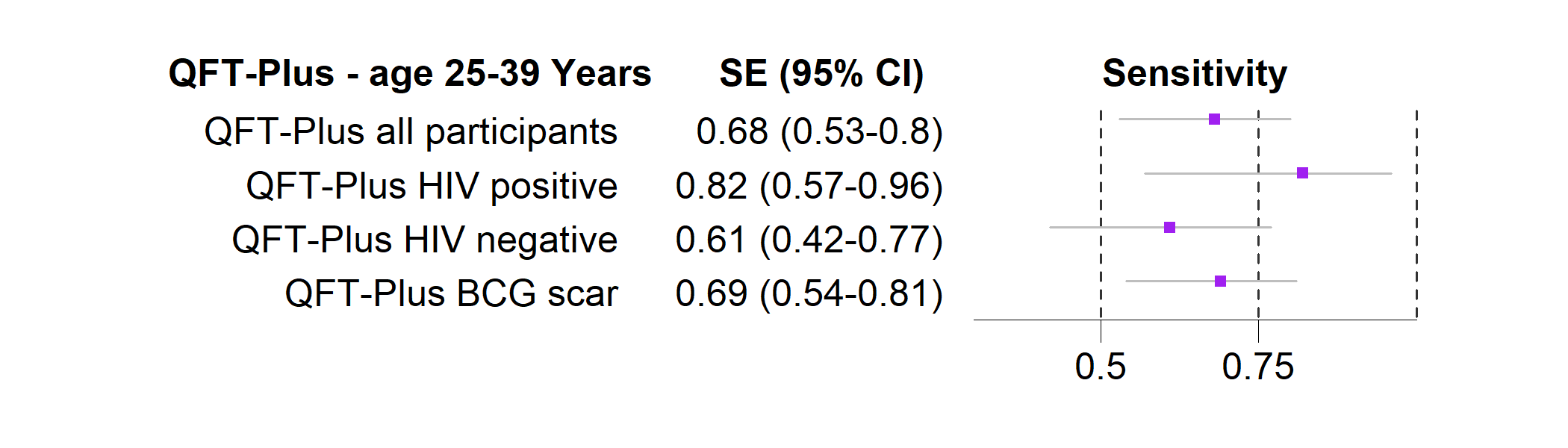

Supplement: SUPPLEMENTARY FIGURE S5 — Accuracy of QFT-Plus among participants aged 18–24 years. [file Image_5.TIFF]

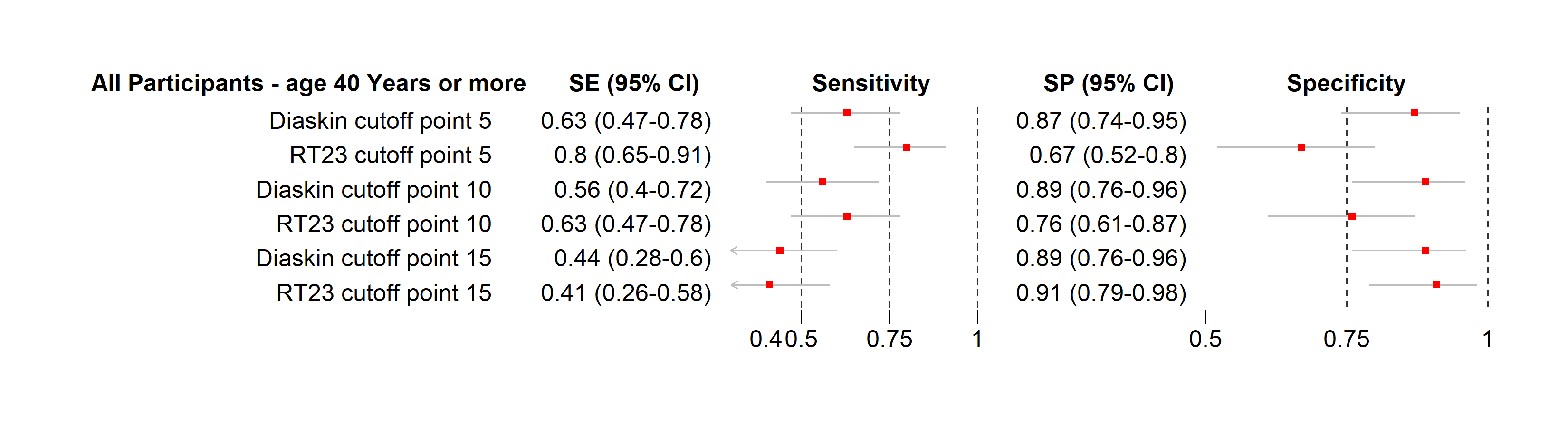

Supplement: SUPPLEMENTARY FIGURE S6 — Accuracy of QFT-Plus among participants aged 25–39 years. [file Image_6.TIFF]

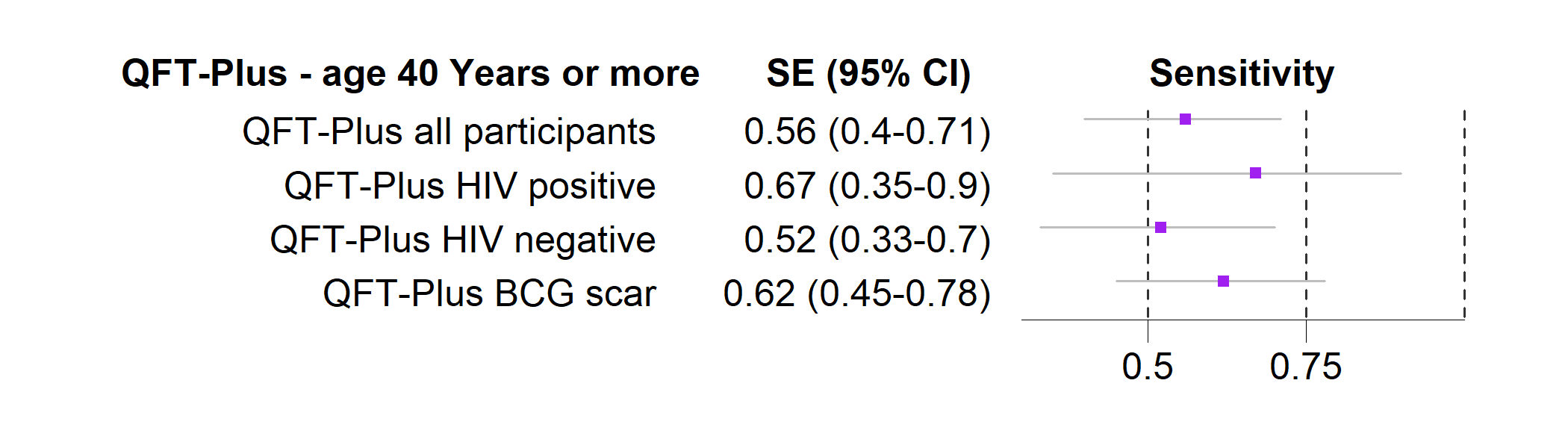

Supplement: SUPPLEMENTARY FIGURE S7 — Accuracy of QFT-Plus among participants aged 40 years or older. [file Image_7.TIFF]

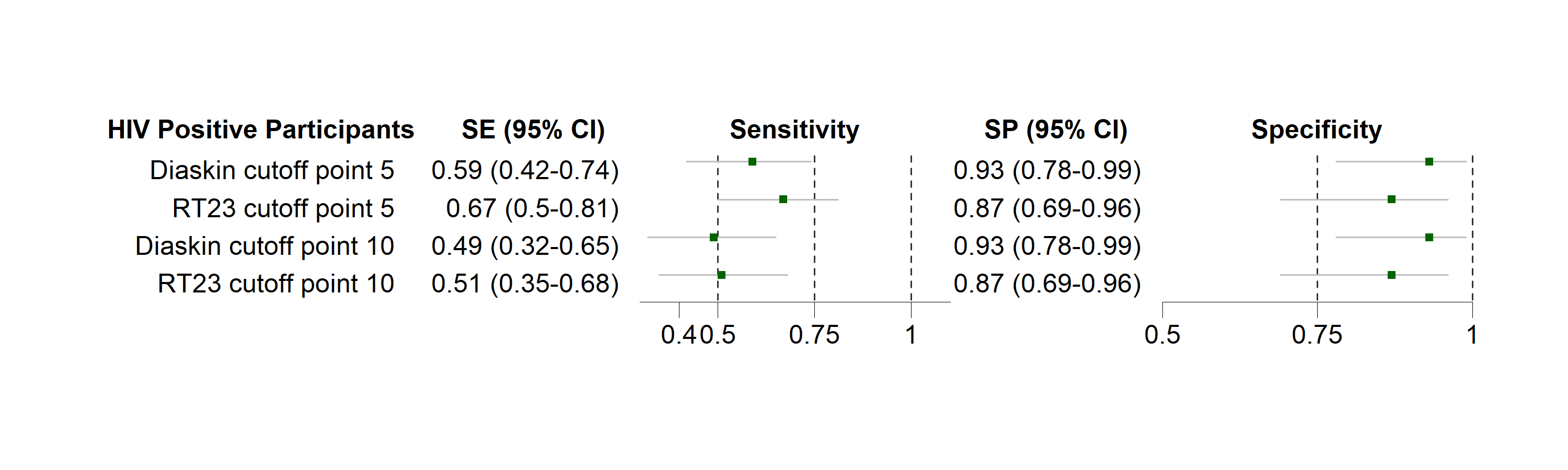

Supplement: SUPPLEMENTARY FIGURE S8 — Accuracy of Diaskintest and PPD Rt-23 among HIV-positive participants. [file Image_8.TIFF]

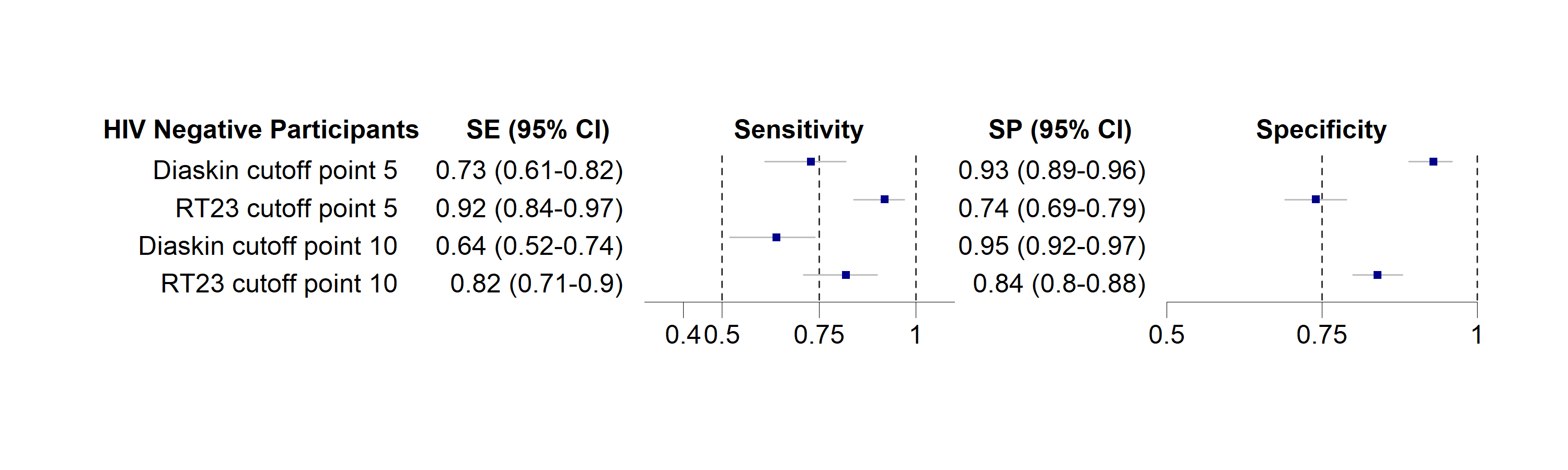

Supplement: SUPPLEMENTARY FIGURE S9 — Accuracy of Diaskintest and PPD Rt-23 among HIV-negative participants. [file Image_9.TIFF]
